# *Brpf1* Haploinsufficiency Impairs Dendritic Arborization and Spine Formation, Leading to Cognitive Deficits

**DOI:** 10.3389/fncel.2019.00249

**Published:** 2019-06-04

**Authors:** Yan Su, Junhua Liu, Baocong Yu, Ru Ba, Chunjie Zhao

**Affiliations:** Key Laboratory of Developmental Genes and Human Diseases, Ministry of Education, School of Medicine, Southeast University, Nanjing, China

**Keywords:** *Brpf1*, intellectual disability, dendrite arborization, spine morphogenesis, synaptic formation

## Abstract

Haploinsufficiency of the bromodomain and PHD finger-containing protein 1 (*BRPF1*) gene causes intellectual disability (ID), which is characterized by impaired intellectual and cognitive function; however, the neurological basis for ID and the neurological function of *BRPF1* dosage in the brain remain unclear. Here, by crossing *Emx1-cre* mice with *Brpf1^fl/fl^* mice, we generated *Brpf1* heterozygous mice to model *BRPF1*-related ID. *Brpf1* heterozygotes showed reduced dendritic complexity in both hippocampal granule cells and cortical pyramidal neurons, accompanied by reduced spine density and altered spine and synapse morphology. An *in vitro* study of *Brpf1* haploinsufficiency also demonstrated decreased frequency and amplitude of miniature EPSCs that may subsequently contribute to abnormal behaviors, including decreased anxiety levels and defective learning and memory. Our results demonstrate a critical role for *Brpf1* dosage in neuron dendrite arborization, spine morphogenesis and behavior and provide insight into the pathogenesis of *BRPF1*-related ID.

## Introduction

Neurons display highly specialized and polarized morphology with distinct regions optimized for their functional roles (Craig and Banker, 1994; Caceres et al., 2012). Typically, a neuron has several dendrites that act as information input centers and a single long axon that is responsible for information output (DeFelipe and Farinas, 1992; Arikkath, 2012; Wang et al., 2014). During the development of cortical networks, the arborization of dendrites and the formation of the dendritic spines of pyramidal neurons are critical for neural circuit assembly (Hausser et al., 2000; Branco et al., 2010; Lavzin et al., 2012). Dendritic spines, the small protrusions on the dendrites, make up the postsynaptic component of most excitatory synapses (Harris, 1999; Sheng and Hoogenraad, 2007; Bourne and Harris, 2008). Previous studies have shown that both cell intrinsic and extrinsic cues, such as secreted signaling molecules (Yacoubian and Lo, 2000), transcription factors (Redmond et al., 2002; Bonini et al., 2011) and postsynaptic density proteins (Charych et al., 2006), contribute to the development of dendritic arborization and spines. Recently, epigenetic modulators, such as histone acetylases (HATs) and histone deacetylases (HDACs), have emerged as regulators of chromatin remodeling that control access to the genes that regulate dendritic growth (Gaub et al., 2010). Neurological disorders, such as mental retardation and cognitive disability, have been shown to be closely associated with defects in dendritic architecture and spine formation (Huttenlocher, 1974; Hutsler and Zhang, 2010; Penzes et al., 2011; Pavlowsky et al., 2012; Yu and Lu, 2012). However, the molecular mechanisms underlying circuit formation remain unclear.

Bromodomain and PHD finger-containing protein 1 (BRPF1) has been demonstrated to act as a multivalent chromatin reader, forming tetrameric complexes with three histone acetyltransferases, MOZ (monocytic leukemia zinc finger protein), MORF (MOZ-related factor), and HBO1 (HAT bound to ORC1), to stimulate acetyltransferase activity and restrict substrate specificity (Doyon et al., 2006; Ullah et al., 2008; Lalonde et al., 2013). *Brpf1* is highly conserved during evolution (Yang and Ullah, 2007). Previous studies have shown that *Brpf1* functions upstream of Hox genes to regulate the patterning of the vertebrate head (Laue et al., 2008; Hibiya et al., 2009). *Brpf1* mutant zebrafish display craniofacial defects, with shifts in the segmental identities of craniofacial arches (Laue et al., 2008). Inactivation of *Brpf1* in medaka alters craniofacial and caudal skeletons (Hibiya et al., 2009), which suggests a critical role for *Brpf1* during development. *Brpf1* is also highly expressed in the developing brain in mice (You et al., 2014). Constitutive deletion of *Brpf1* leads to embryonic lethality with severe abnormalities, including defects in neural tube closure (You et al., 2015b). The forebrain-specific deletion of *Brpf1* results in early postnatal lethality and growth retardation (You et al., 2015c). Viable mice exhibit partial agenesis of the corpus callosum, hypoplasia of the dentate gyrus (DG) and behavioral abnormalities (You et al., 2015a,c), which indicates that *Brpf1* is required for the development of the telencephalon.

Recently, *Brpf1* was reported as a candidate gene for intellectual disability (ID) (Mattioli et al., 2017; Yan et al., 2017). High-throughput sequencing in ID cohorts has identified *de novo* or inherited monoallelic *BRPF1* mutations. Patients carrying heterozygous mutations of *BRPF1* suffer from a neurodevelopmental disorder characterized by congenital hypotonia, facial dysmorphisms, global developmental delay, and ID (Yan et al., 2017), which suggests that *Brpf1* may regulate brain development in a dosage-dependent manner. To elucidate the mechanism underlying *BRPF1*-related ID and assess the effect of *BRPF1* haploinsufficiency on pyramidal maturation and behavior, we generated *Brpf1* heterozygote mice by crossing *Emx1-cre* with *Brpf1*^fl/fl^.

We found that *Brpf1* haploinsufficiency led to reduced dendritic complexity and a decreased number of dendritic spines. Moreover, dendritic spine morphology was altered, with longer spines in the haploinsufficient group than in the control group. Physiological analysis showed decreased excitatory synaptic transmission that subsequently led to defective learning and memory. These findings strongly demonstrate that *Brpf1* dosage is very important during dendritic arborization and spine formation. Our study will help to elucidate the neurobiological mechanisms underlying ID.

## Materials and Methods

### Animals

*Brpf1*^fl/fl^ mice were obtained from the European Conditional Mouse Mutagenesis Program (EUCOMM, project 40402). *Emx1-Cre* mice were purchased from The Jackson Laboratory (Stock: 005628). A dorsal telencephalon-specific disruption of *Brpf1* was accomplished using *Emx1-Cre*, which drives recombination in both progenitor and projection neurons in the dorsal telencephalon. Through Emx1-Cre mediated recombination, the loxP-flanked region spanning exons 4–6 in *Brpf1* locus was deleted. Primer pairs 5′-TGTGCCCTGTAGAGTGTTGC-3′ and 5′-GCCTTGAGTGGCACAACATA-3′ which amplify a 227-bp band for wild-type and a 440-bp band for *Brpf1*^fl/fl^ were used. The *Emx1-cre*; *Brpf1*^fl/+^ mice were referred to as *Brpf1* heterozygous mice (HT), and the *Emx1-cre*; *Brpf1*^fl/fl^ mice were referred to as *Brpf1* conditional knock-out mice (cKO). The *Brpf1*^fl/fl^ mice were referred to as wild-type mice (WT). Mice used for behavioral testing were maintained on a C57BL/6 background, and for histological analysis, mice were maintained on an ICR background. The day of vaginal plug detection was considered embryonic day 0.5 (E0.5), and the day of birth was considered postnatal day 0 (P0). All animals were bred in the animal facility at Southeast University. No obvious differences were detected between the sexes. All experiments were performed according to the approved guidelines of Southeast University.

### Behavioral Tests

Male mice aged 2–4 months were used for behavioral assays, which were conducted during the light phase. The experimenters were blinded to the genotype of each mouse during all tests and data analyses. The tests were performed in the following sequence: open-field test, elevated zero maze (O-maze) test, Morris water maze test and fear conditioning test.

### Open-Field Test

For the open-field test, each mouse was placed in a square chamber (40 × 40 cm) as previous reported (Wu et al., 2014). The movement of the mice was recorded using a Unibrain Fire-i digital camera. Locomotor and exploratory activity was assessed by the number of basic movements, whereas the proportion of time spent in the center of the enclosure was used as a measure of anxiety. Data were collected for 30 min. The chamber was cleaned between each trial.

### Elevated O-Maze Test

The O-maze equipment consisted of a 6-cm-wide ring with an outer diameter of 45 cm containing two equal closed sections and two open sections. The entire ring was elevated to a height of 100 cm. Mice were placed at the boundary between the walled and unwalled sections, facing the unwalled side. The time spent in the open and closed arms was recorded for 10 min.

### Morris Water Maze Test

A circular pool 120 cm in diameter was used, with the water maintained at 23.0 ± 0.5°C and made opaque by white nontoxic paint (Jiangsu Taibai, China). Depending on the test session, 4 distinct high-contrast posters, which served as spatial cues, were attached to the pool wall. A round platform 10 cm in diameter was positioned 0.5 cm below the water surface. EthoVision software (Noldus) was used to track the mice in the maze and analyze the data. Mice were first subjected to 1 day (d) of visible platform training. After the visible platform training session, a mouse was released into the pool, facing the pool wall, at pseudorandom starting positions. After reaching the platform, the mouse was allowed to stay on the platform for 15 s before being moved back to the home cage; if the mouse failed to find the platform within 60 s, it was manually placed on the platform for 15 s. Each mouse received four trials daily of the above-described training sessions. The training sessions lasted for 8 days. Twenty-four hours after the last training session (day 9), the platform was removed, and 60-s probe trials were performed, with the mice being released at the center of the pool. The daily averaged latency to reach the platform for each mouse was used to assess learning progress. The duration that mice stayed in each quadrant in the probe test was used to evaluate the spatial memory.

### Fear Conditioning Test

Fear conditioning tests were performed following a standard training protocol (Ugo Basile). Mice were placed in a Plexiglas shock chamber and were allowed to explore the chamber for 180 s. A 2800 Hz tone was then sounded for 30 s as the conditioning stimulus; during the last 1 s of the tone, a 0.3 mA foot shock was delivered. The cue-shock training was then repeated three times. All tests were performed 24 h after training. For contextual conditioning, mice were placed in the same chamber used for the training test, and freezing times were recorded for 8 min. For cued conditioning, mice were placed in a novel chamber for 3 min; the test lasted 8 min after the tone used in the training test was played. The freezing time during the pretone and posttone periods was recorded.

### Immunostaining and HE Staining

Brains were fixed by transcardial perfusion with cold 4% paraformaldehyde (PFA) after the animals were deeply anesthetized with pelltobarbitalum natricum (50 mg/kg). The brains were then post-fixed overnight at 4°C, cryoprotected in 30% sucrose, embedded in optimum cutting temperature (OCT) compound and stored at -70°C until further use. The brains were cryosectioned into 25-μm-thick sections using a Leica CM 3050S cryostat. Immunostaining was then performed as previously reported (Tian et al., 2012; Liu et al., 2018). The following antibodies were used: rabbit anti-Tbr1 (T-box brain gene 1) (Millipore, AB10554, 1:1000); rat anti-Satb2 (special AT-rich sequence binding protein 2) (Santa Cruz Biotechnology, sc31876, 1:1000); and rat anti-Ctip2 (COUP-TF interacting protein 2) (Abcam, ab18465, 1:1500); rabbit anti-GFAP (Glial Fibrillary Acidic Protein) (Sigma, G9269, 1:1000). Alexa Fluor 633 goat anti-mouse IgG (Molecular Probes, A21050, 1:500), Alexa Fluor 488 goat anti-rabbit IgG (Molecular Probes, A11008, 1:500), and Alexa Fluor 546 goat anti-rat IgG (Molecular Probes, A11081, 1:500) were used as secondary antibodies. For HE staining, frozen sections were stained with hematoxylin and eosin as previously reported (Yunus et al., 2014), dehydrated through an ascending ethanol series, cleaned with xylene, and coverslipped with premount (LEAGENE, China). For morphometric analysis, at least three brains were analyzed for each condition in parallel experiments. Both hemispheres of at least three matching sections from each brain were used for the measurements. The quantification of the thickness of corpus callosum was manually measured using ImageJ software (NIH).

### Electrophysiological Recordings

Mice aged P28-P35 were used for electrophysiological experiments. Mice were first anesthetized with an intraperitoneal injection of pentobarbital (100 mg/kg body weight) and decapitated. The brain was then quickly removed and immersed in ice-cold oxygenated artificial CSF (ACSF) containing the following (in mM): 125 NaCl, 2.5 KCl, 1.25 NaH_2_PO_4_, 26 NaHCO_3_, 1 CaCl_2_, 6 MgCl_2_, and 10 glucose. Coronal slices (400 μm) were generated using a vibrating microtome (VT1000; Leica Microsystems) and incubated in a holding chamber at 32–35°C for 30 min, followed by continued incubation at room temperature for at least 1 h before physiological recordings. A slice was then transferred to a recording chamber attached to the microscope stage and completely submerged in ACSF containing the following (in mM): 125 NaCl, 26 NaHCO_3_, 2.5 KCl, 1.25 NaH_2_PO_4_, 4 CaCl_2_, 4 MgCl_2_, and 10 glucose, pH 7.4 (bubbled with 95% O_2_/5% CO_2_). ACSF was perfused through the recording chamber at 3 ml/min at 32°C.

Whole-cell patch-clamp recordings were obtained from visually identified neurons using an infrared differential interference contrast video microscopy system. For current and voltage clamp recordings, the pipette solution contained the following (in mM): 125 potassium D-gluconate, 8 NaCl, 0.2 EGTA, 10 HEPES, 2 Mg-ATP, 0.3 Na-GTP, and 0.1% Biocytin. Patch electrodes (3–6 MΩ) were pulled from borosilicate glass capillaries (outer diameter, 1.5 mm). Series resistances were usually 15–30 MΩ upon break-in and were compensated by 70%, and only cells with stable series resistance (20% change throughout the recording) were used for analysis. Data were collected using an Axon patch 700B amplifier (Molecular Devices), low-pass filtered at 2 kHz and digitally sampled at 10 kHz online, and analyzed offline with Clampfit software (Molecular Devices). To characterize the intrinsic membrane properties of neurons, current-clamp recordings were made, and hyperpolarizing and depolarizing current steps of 400 ms duration were injected at 40 pA increments at 0.1 Hz. The following parameters were measured to characterize neuronal membrane properties: the resting membrane potential was recorded immediately after the rupture of the neuronal membrane and the input resistance was determined by measuring the voltage change in response to a hyperpolarizing current pulse. The action potential current threshold was defined as the first 400 ms rectangular current injection that elicited a spike. To record spontaneous miniature EPSCs (mEPSCs), slices were recorded with bath application of bicuculline (BMI, Sigma-Aldrich, 14343, 10 μM) and tetrodotoxin (TTX, MCE, 1 μM) to block GABA receptor-mediated inhibitory currents and action potential-dependent synaptic transmission, respectively. mEPSCs were analyzed using the Mini Analysis Program (Version 6.0.3, Synaptosoft), and all events were detected above a threshold of 5 pA.

### Golgi Staining and Morphometric Analysis

Golgi-Cox staining of brains was performed using the FD Rapid Golgi Stain Kit (FD Neurotechnologies). Brains were obtained from adult mice and impregnated with Golgi-Cox solution at room temperature in the dark for 5 days. Brains were then transferred to solution C for 2 days, followed by sectioning at 120 μm with a vibrating microtome (VT1000; Leica Microsystems) and staining according to the manufacturer’s protocol. At least four brains per genotype were impregnated. Pyramidal neurons in layer V of the neocortex and granule cells in the DG were captured by a confocal microscope (Olympus Fluoview FV1000). Each neuron was manually traced using ImageJ software (NIH). Dendritic complexity was assessed using Sholl analysis to examine the number of dendritic intersections per 10-μm concentric radial interval from the cell body. The significance of the differences in complexity was determined using GraphPad Prism software by two-way ANOVA (genotype and circle radius as factors) with Bonferroni *post hoc* test. Values of *P* < 0.05 were considered statistically significant.

To calculate the spine density of Golgi-stained neurons in the cortex, the length of apical dendrite secondary branches was traced, the exact length of the dendritic segment was calculated, and the number of spines along that length was counted. The accurate spine length was manually measured using ImageJ software. The data were analyzed statistically by one-way ANOVA followed by Bonferroni *post hoc* analysis. Values of *P* < 0.05 were considered statistically significant.

### Primary Neuron Culture, Immunostaining, and Quantification

Primary cortical neurons were prepared from *Brpf1^fl/fl^* (WT), *Emx1-cre*; *Brpf1^fl/+^* (HT), and *Emx1-cre*; *Brpf1^fl/fl^* (cKO) E16.5 brains according previously described (Yu et al., 2014). The dorsal telencephalon was dissected from individual embryos in ice-cold HBSS (Thermo Fisher Scientific, 14170112) and digested with 0.125% trypsin (Thermo Fisher Scientific, 25200) for 8 min at 37°C, followed by neutralization with 2 ml of DMEM (Thermo Fisher Scientific, 11330032) supplemented with 10% FBS (Thermo Fisher Scientific, 1047028), 1% GlutaMAX-1 (Thermo Fisher Scientific, 35050061), and 0.2% penicillin/streptomycin (Thermo Fisher Scientific, 15070063) as previous reported (Yang et al., 2017; Shen et al., 2019). Neurons were then dissociated by pipetting, and the suspensions were centrifuged at 1000 rpm for 5 min. The cells were then resuspended, plated at a density of 2 × 10^4^ cells/cm^2^, and cultured in neurobasal medium (Thermo Fisher Scientific, 10888022) supplemented with 2% B27 (Thermo Fisher Scientific, 17504044), 1% GlutaMAX-1 and 0.2% penicillin/streptomycin.

Neurons were cultured for 5 days *in vitro* (DIV) and 14 DIV to quantify axonal and dendritic growth, respectively (Wang et al., 2016). Neurons were fixed in PBS containing 4% paraformaldehyde, permeabilized with 0.1% Triton X-100, and blocked with 10% normal goat serum in PBS for 1 h at room temperature. For immunostaining, neurons were incubated overnight at 4°C with rabbit anti-Tau (Abcam, ab64193, 1:500) and mouse anti-MAP2 (Chemicon, MAB378, 1:1000) primary antibodies, followed by incubation with secondary fluorophore-conjugated antibodies.

The quantification of neuronal dendritic complexity and axon length was performed using the same method described for Golgi-stained neurons.

### Electron Microscopy

Brains were fixed by transcardial perfusion with cold 4% PFA after the animals were deeply anesthetized. The dissected tissue from the CA3 region of the hippocampus was then fixed in 2% glutaraldehyde and 2% paraformaldehyde in 0.1 M phosphate buffer overnight at 4°C and post-fixed in 1% osmium tetroxide in phosphate buffer. After washing five times for 30 min each time in phosphate buffer, the samples were dehydrated through a series of ethanol washes. After further dehydration in propylene oxide, the tissues were embedded in low-viscosity embedding resin and sectioned into 60-nm sections with an ultramicrotome (Reichert-Jung) fitted with a 45° diamond knife. Ultrathin sections (60 nm) were stained with uranyl acetate followed by lead citrate for 15 min. Images were obtained on a JEM-1010 transmission electron microscope at an accelerating voltage of 80 kV. The synaptic length was assessed as the length of the postsynaptic density. The synaptic area was assessed as the area of the postsynaptic density. The synaptic cleft was measured as the gap between the presynaptic and postsynaptic membranes in digitally magnified images.

### Microscopy and Image Analysis

Immunostained-sections and Golgi-stained sections were viewed under a confocal microscope (Olympus FV1000), and the images were collected and analyzed using FV10-ASW image analysis software. HE staining sections were viewed and collected under a fluorescence microscope (Olympus DP71).

### Statistical Analysis

Statistical significance was determined using two-tailed, unpaired Student’s *t* tests for two-population comparisons for the open field test, the elevated zero maze test, the cued and conditioning fear tests, immunostaining, HE staining and electrophysiological experiments. One-way ANOVA with Bonferroni *post hoc* test was used for multiple comparisons for electron microscopy. The quantitative results are expressed as the mean ± the standard error of the mean (SEM). ^∗^*P* < 0.05, ^∗∗^*P* < 0.01, and ^∗∗∗^*P* < 0.001. Values of *P* < 0.05 were considered statistically significant. All the data were graphically represented with GraphPad Prism software.

## Results

### Reduced Anxiety Levels and Impaired Learning and Memory in *Brpf1* Heterozygotes

**Figure 1:**
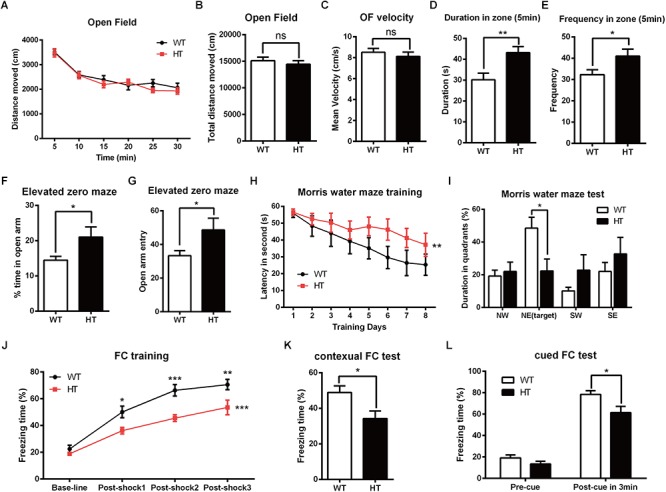
*Brpf1* HT mice display decreased anxiety levels and impaired learning and memory. **(A)** The general activity levels of *Brpf1* HT mice are similar to those of WT mice [WT: *n* = 10; HT: *n* = 10; two-way ANOVA (effect of genotype: *P* = 0.2923) with Bonferroni *post hoc* test]. **(B)** The total distance traveled in 30 min is similar between *Brpf1* WTs and HTs in the open field test (WT: *n* = 10; HT: *n* = 10; *P* = 0.4909). **(C)** *Brpf1* HTs show a comparable mean velocity to that of WTs in the open field test (WT: *n* = 10; HT: *n* = 10; *P* = 0.4874). **(D)** The *Brpf1* HT mice show a 42.8% increase in the duration spent in the center zone within the first 5 min of the open field test by comparison of the time spent by *Brpf1* HTs and WTs in the center zone (WT: *n* = 11; HT: *n* = 10; *P* = 0.0077). **(E)** The *Brpf1* HT mice travel much more frequently to the center zone within the first 5 min of the open field test (WT: *n* = 11; HT: *n* = 10; *P* = 0.0402). **(F,G)** In the elevated zero maze test, the *Brpf1* HT mice spend significantly more time than the WT mice in the open arms (WT: *n* = 14; HT: *n* = 11; *P* = 0.0311) and make more entries into the open arms (WT: *n* = 14; HT: *n* = 11; *P* = 0.0411). **(H)** The Morris water maze test demonstrates spatial learning disabilities in *Brpf1* HT mice [WT: *n* = 8; HT: *n* = 10; two-way ANOVA (effect of genotype: *P* = 0.0004) with Bonferroni *post hoc* test]. **(I)** The quantification of the time spent in each quadrant during the probe trial [WT: *n* = 8; HT: *n* = 10; NE (target): *P* = 0.0188]. **(J)** *Brpf1* HTs exhibit decreased freezing time during fear conditioning training [WT: *n* = 9; HT: *n* = 9; two-way ANOVA (effect of genotype: *P* < 0.0001) with Bonferroni *post hoc* test, *P* < 0.05 at post-shock 1, post-shock 2, and post-shock 3]. **(K)** *Brpf1* HTs exhibit decreased freezing time in the contextual fear conditioning test (WT: *n* = 9; HT: *n* = 9; *P* = 0.0198). **(L)** In the cued fear conditioning test, freezing time after cue presentation is decreased in *Brpf1* HTs compared to WTs (WT: *n* = 9; HT: *n* = 9; *P* = 0.0227). Two-tailed, unpaired Student’s *t*-test, ^∗^*P* < 0.05, ^∗∗^*P* < 0.01, ^∗∗∗^*P* < 0.001.

To investigate the impact of *Brpf1* haploinsufficiency on behavior in mice, we mainly focused on the role of *Brpf1* in the dorsal telencephalon and related behaviors. The *Emx1-cre* (Gorski et al., 2002) line was then crossed with *Brpf1^fl/fl^* mice to generate *Brpf1* heterozygous mice (*Emx1-cre*; *Brpf1^fl/+^*, abbreviated HTs) and *Brpf1* cKO mice (*Emx1-cre*; *Brpf1^fl/fl^*, abbreviated cKOs). As previously reported, *Emx1-cre* medicate recombination occurs as early as E10.5 during the development of the telencephalon, thus *Brpf1* was disrupted in both telencephalic excitatory glutamatergic neurons and astroglias, while GABAergic interneurons arising from the ventral ganglionic eminence were not affected. Due to the lethality of adult cKO mice in a C57BL/6 background, we only compared HTs and *Brpf1^fl/fl^* wild-type (WT) mice. Males aged 2 to 4 months were used in our behavioral analyses to eliminate the possible impact of the estrous cycle on the behavioral performance of the rodents (Markus and Zecevic, 1997; Jasnow et al., 2006; van Goethem et al., 2012). The first open field test was carried out to evaluate spontaneous motor ability and anxiety. As shown in Figure 1A, the total distance traveled during each 5-min duration was similar. The total distance traveled in a period of 30 min was comparable between the HTs and WTs (Figure 1B), and no obvious differences in the mean velocity were detected (Figure 1C), which reflects that locomotor activity was unaffected. Since the behavior within the first 5 min of the open field test best reflects anxiety levels, we then measured the duration spent in the center zone during the first 5 min. *Brpf1* HTs spent more time in the center zone than the WTs did (Figure 1D). Identical results were observed for the frequency of entering the center zone (Figure 1E). This result indicates that the anxiety levels of *Brpf1* HTs were possibly decreased. The elevated zero maze test showed that the time spent in the open arms and the frequency of entering the open arms were significantly greater for the HTs than for the WTs (Figure 1F,G), further demonstrating that *Brpf1* HTs were less anxious.

To evaluate spatial learning and memory, we next conducted the Morris water maze test (Morris, 1984). Mice were trained according to an 8-days training protocol to find a hidden platform submerged under water. As shown in Figure 1H, *Brpf1* HTs spent more time finding the platform, suggesting a significant defect in spatial learning throughout the training period. Spatial memory was then assessed 24 h after the training period by allowing the mice to search the pool from which the platform had been removed. *Brpf1* HTs spent less time in the target quadrant where the platform was previously located (Figure 1I), which demonstrates limited spatial memory abilities. We next evaluated memory deficits through contextual and cued fear conditioning. During the training phase, after being conditioned to electric shocks, *Brpf1* HTs showed decreased freezing time compared to that of the WTs, which demonstrates that fear learning was impaired (Figure 1J). When mice were returned to the same context 24 h after training, *Brpf1* HTs showed significantly less freezing time than that of the WTs (Figure 1K). In the cued fear conditioning test, *Brpf1* HTs also showed reduced freezing time (Figure 1L). Together, these behavioral tests indicate that *Brpf1* heterozygote mice have defects in learning and memory accompanied by decreased anxiety-related behaviors, indicating that *Brpf1* functions in cognitive behaviors in a dosage-dependent manner in mice.

### Excitatory Synaptic Transmission Is Reduced After *Brpf1* Deletion

**Figure 2:**
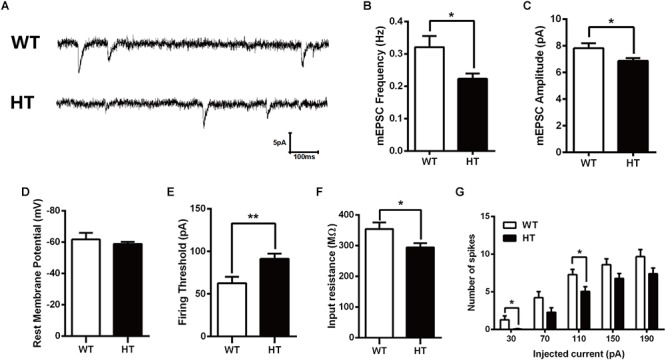
Decreased frequency and amplitude of mEPSCs in pyramidal neurons from *Brpf1* HT mice. **(A)** Representative traces of mEPSCs recorded in hippocampal CA1 pyramidal neurons of P30 WTs (top) and *Brpf1* HTs (bottom). Note that the frequency and amplitude of mEPSCs recorded in excitatory neurons from *Brpf1* HT mice are significantly decreased. **(B)** Statistical comparison of the mean mEPSC frequency in excitatory neurons from *Brpf1* WT and HT mice (WT: *n* = 16; HT: *n* = 17; *P* = 0.0142). **(C)** Statistical comparison of the mean mEPSC amplitude in excitatory neurons from *Brpf1* WT and HT mice (WT: *n* = 16; HT: *n* = 17; *P* = 0.0277). **(D)** *Brpf1* HT cells show comparable resting membrane potentials to those of WT cells (WT: *n* = 16; HT: *n* = 17; *P* = 0.0926). **(E)** The mean current threshold required to elicit a spike is markedly increased in *Brpf1* HT pyramidal neurons (WT: *n* = 16; HT: *n* = 17; *P* = 0.0054). **(F)** The mean input resistance is significantly reduced (WT: *n* = 16; HT: *n* = 17; *P* = 0.0213). **(G)** The number of spikes is displayed against depolarizing current steps of increasing amplitude. Five 400-ms depolarizing currents ranging from 30 to 190 pA at 40-pA intervals were injected. Notably, *Brpf1* HT pyramidal neurons fire fewer action potentials than control neurons in response to the same amount of current (WT: *n* = 18; HT: *n* = 18; 30 pA: *P* = 0.0230; 110 pA: *P* = 0.0245). Two-tailed, unpaired Student’s *t*-test, ^∗^*P* < 0.05, ^∗∗^*P* < 0.01, ^∗∗∗^ *P* < 0.001.

Alterations in learning and memory are closely associated with synaptic dysfunction (Volk et al., 2015). To assess the functional consequence of *Brpf1* ablation on synaptic transmission, whole-cell patch-clamp recording was performed in CA1 pyramidal cells from acute slices of mice. To examine baseline spontaneous activity, we compared the frequency and amplitude of spontaneous miniature EPSCs (mEPSCs). Since *Brpf1* was deleted in excitatory but not inhibitory neurons, only mEPSCs in excitatory neurons were then recorded with bath application of BMI (10 μM) and TTX (1 μM) to block GABA receptor-mediated inhibitory currents and action potential-dependent synaptic transmission. As shown in Figure 2A,B, the mean frequency of mEPSCs in *Brpf1* HTs was strongly decreased compared with that in WT mice. Moreover, the mean amplitude of mEPSCs was also reduced (Figure 2C). These data suggest that synaptic transmission is reduced by *Brpf1* haploinsufficiency.

To investigate the effect of *Brpf1* haploinsufficiency on the maturation and excitability of pyramidal neurons, we next tested the membrane properties of CA1 pyramidal neurons. After contacting the cell membrane, the resting membrane potential was immediately recorded, as shown in Figure 2D. The mean resting potential was comparable between HTs and WTs, while the mean action potential current threshold was significantly higher in the HTs than in the WTs (Figure 2E). Input resistance was then calculated by measuring the voltage deflection in response to a hyperpolarizing current pulse. *Brpf1* haploinsufficiency led to a significant decrease in the mean input resistance of pyramidal neurons (Figure 2F), consistent with the increase in firing current threshold. As a result, *Brpf1* heterozygous neurons fired fewer action potentials than WT neurons when responding to the same current injection (Figure 2G). Collectively, these data show that *Brpf1* haploinsufficiency causes reduced synaptic transmission and decreased cell excitability, which may contribute to abnormal behaviors.

### *Brpf1* HTs Exhibit a Slightly Thinner Corpus Callosum

**Figure 3:**
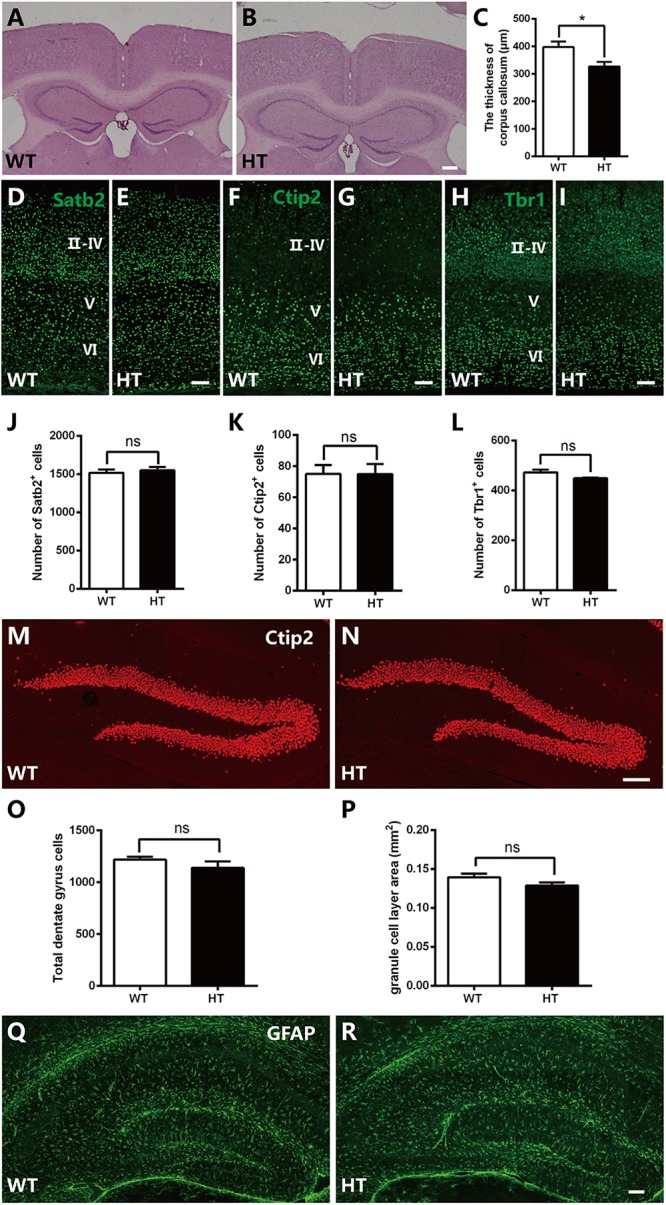
*Brpf1* HT mice exhibit a slightly thinner corpus callosum with gross comparable brain morphology. **(A,B)** Representative coronal brain sections of HE staining in *Brpf1* WT mice **(A)** and HT mice **(B)** at the adult stage. **(C)** Quantitative analysis of the thickness of corpus callosum at the adult stage (WT: *n* = 3 brains; HT: *n* = 4 brains; *P* = 0.0422). **(D–I)** Representative coronal brain sections immunostained with anti-Satb2 **(D,E)**, anti-Ctip2 **(F,G)**, anti-Tbr1 **(H,I)** antibodies from *Brpf1* WT mice **(D,F,H)** and HT mice **(E,G,I)** at the adult stage. **(J–L)** Quantitative analysis of cortical lamination at the adult stage. The number of Satb2-positive (**J**, WT: *n* = 4 brains; HT: *n* = 4 brains; *P* = 0.6240), Ctip2-positive (**K**, WT: *n* = 4 brains; HT: *n* = 4 brains; *P* = 0.9908), and Tbr1-positive (**L**, WT: *n* = 4 brains; HT: *n* = 3 brains; *P* = 0.1446) cells are similar between *Brpf1* WT and HT mice. **(M,N)** Representative fluorescent images of hippocampal DG sections immunostained with anti-Ctip2 antibodies from *Brpf1* WT mice **(M)** and HT mice **(N)**. **(O)** The total number of DG granule cells is comparable between *Brpf1* WT mice and HT mice (WT: *n* = 3 brains; HT: *n* = 4 brains; *P* = 0.3455). **(P)** Quantitative analysis of granule cell layer area shows no noticeable difference (WT: *n* = 6 brains; HT: *n* = 6 brains; *P* = 0.1189). **(Q,R)** Representative fluorescent images of hippocampal sections immunostained with anti- GFAP antibodies from *Brpf1* WT mice **(Q)** and HT mice **(R)**. Scale bar: **(A,B)**: 200 μm; **(D–I,M,N,Q,R)**: 100 μm. Two-tailed, unpaired Student’s *t*-test, ^∗^*P* < 0.05, ^∗∗^ *P* < 0.01, ^∗∗∗^*P* < 0.001.

*Brpf1* has been reported to be required for cell proliferation and neuronal migration during cortical development (You et al., 2015a,c). To examine whether the cognitive and synaptic transmission defects observed in *Brpf1* HTs were caused by morphological deficiencies, we compared the overall organization of adult brains. HE staining showed that the cellular organization and regional anatomy between the brains of *Brpf1* HTs and WTs were indistinguishable. No obvious defects were observed in the hippocampus or the DG. However, the corpus callosum was slightly thinner in *Brpf1* HTs, as observed in coronal sections rostrally to caudally (Figure 3A,B and Supplementary Figure S1). The quantification results revealed that the thickness of corpus callosum was decreased 17.7% after *Brpfl* haploinsufficiency (Figure 3C). To determine whether there was any alteration in cortical lamination in adult brains, we performed immunostaining for the transcription factors Satb2, Ctip2, and Tbr1, which are established markers of callosal projection neurons, subcerebral projection neurons, and corticothalamic neurons, respectively (Arlotta et al., 2005; Molyneaux et al., 2007; Fishell and Hanashima, 2008). As shown in Figure 3D–I, the distribution of the subtypes of cortical neurons seemed normal. Quantification revealed no significant differences in the number of Satb2^+^, Ctip2^+^, and Tbr1^+^ neurons between *Brpf1* HT and WT mice (Figure 3J–L), demonstrating that *Brpf1* HTs develop grossly normal cortices. We next assessed granule neurons in the DG by immunostaining with anti-Ctip2 (Figure 3M,N). The number of granule neurons and the area of the granule cell layer in *Brpf1* HTs were comparable to those of WTs (Figure 3O,P). Abnormal formation of synapse or alteration of excitatory synapse function could also be linked to the glial cells dysfunction. We then performed the immunostaining using astrocyte-specific marker GFAP, the results showed that there were no obvious alteration in glial cells organization in these mice (Figure 3Q,R). Taken together, these results suggest that *Brpf1* haploinsufficiency results in a slightly thinner corpus callosum but has no obvious effects on the overall cellular organization of the telencephalon.

### *Brpf1* Haploinsufficiency Leads to Decreased Dendritic Complexity and Abnormal Spine Formation

**Figure 4:**
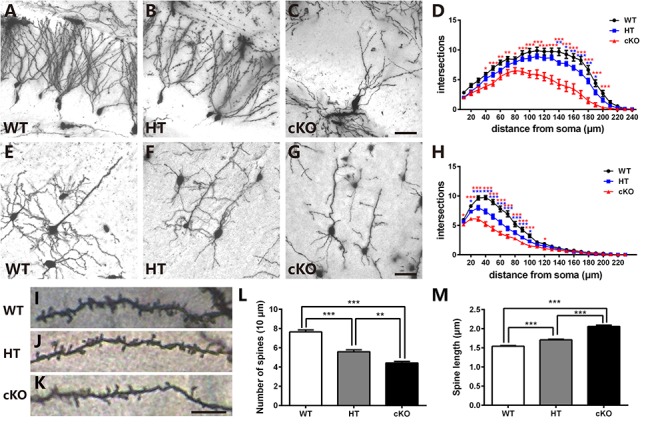
*Brpf1* haploinsufficiency leads to decreased dendritic complexity and altered spine morphology. **(A–C)** Representative Golgi staining images of DG granule cells from *Brpf1* WT **(A)**, HT **(B)**, and cKO **(C)** mice. **(D)** Sholl analysis indicates reduced dendritic complexity of DG granule neurons in *Brpf1* HT and cKO mice [WT: *n* = 26 neurons of 4 mice; HT: *n* = 28 neurons of 4 mice; cKO: *n* = 15 neurons of 4 mice; two-way ANOVA (effect of genotype: *P* < 0.0001) with Bonferroni *post hoc* test: for WT and HT, *P* = 0.0041 at 140 μm, *P* = 0.0194 at 150 μm, *P* = 0.0074 at 160 μm, *P* = 0.0008 at 170 μm, *P* = 0.0042 at 180 μm, *P* = 0.0264 at 190 μm from the cell body (blue asterisk), for WT and cKO, *P* = 0.0111 at 40 μm, *P* = 0.0007 at 50 μm, *P* = 0.0022 at 60 μm, *P* = 0.0028 at 70 μm, *P* = 0.0197 at 80 μm, *P* < 0.0001 at 90–200 μm from the cell body (red asterisk)]. **(E–G)** Representative Golgi staining images of cortical layer V pyramidal neurons from *Brpf1* WT **(E)**, HT **(F)**, and cKO **(G)** mice. **(H)** Sholl analysis shows reduced dendritic complexity of cortical pyramidal neurons in *Brpf1* HT and cKO mice [WT: *n* = 43 neurons of 4 mice; HT: *n* = 41 neurons of 4 mice; cKO: *n* = 52 neurons of 4 mice; two-way ANOVA (effect of genotype: *P* < 0.0001) with Bonferroni *post hoc* test: for WT and HT, *P* = 0.0151 at 20 μm, *P* < 0.0001 at 30–90 μm, *P* = 0.0180 at 100 μm from the cell body (blue asterisk), for WT and cKO, *P* = 0.0383 at 10 μm, *P* < 0.0001 at 20–100 μm, *P* = 0.0366 at 120 μm from the cell body (red asterisk)]. **(I–K)** Representative images of apical dendritic spines of secondary branches from cortical neurons from *Brpf1* WT **(I)**, HT **(J)**, and cKO **(K)** mice are shown. **(L)** Statistical analysis of dendritic spine density reveals decreased spine density in *Brpf1* HT and cKO mice [WT: *n* = 42; HT: *n* = 22; cKO: *n* = 22; one-way ANOVA (*F*_(2,83)_ = 64.34, *P* < 0.0001) with Bonferroni *post hoc* test: *P* < 0.0001 between WT and HT, WT and cKO, *P* = 0.0032 between HT and cKO). **(M)** The quantification of dendritic spine length demonstrates increased spine length in *Brpf1* HT and cKO mice (WT: *n* = 563 spines; HT: *n* = 390 spines; cKO: *n* = 319 spines; one-way ANOVA (*F*_(2,1269)_ = 144.0, *P* < 0.0001) with Bonferroni *post hoc* test: *P* < 0.0001 between WT and HT, WT and cKO, and HT and cKO]. Scale bar: **(A–C, E–G)**: 50 μm; **(I–K)**: 10 μm.

To detect whether the deficits in spatial learning and fear conditioning were associated with aberrant dendritic complexity (Penzes et al., 2011; Powell et al., 2012; Dang et al., 2014), Golgi-Cox staining was performed to measure the dendrites and spines. As shown in Figure 4A, in the WTs, granule cells were fully developed with numerous and complicated dendritic arbors. In contrast, the dendritic trees of granule cells in *Brpf1* HTs were much less complex than those in the WTs (Figure 4B). We also examined the *Brpf1* cKO mice maintained on an ICR background that survived to adulthood. In the cKO mice, the dendrites were significantly impaired with remarkably reduced dendritic complexity (Figure 4C). We then quantified dendritic complexity by counting the intersections where dendrites crossed concentric circles drawn at 10-μm intervals around the neuronal cell bodies. Sholl analysis revealed a significant decrease in the number of intersections in *Brpf1* HT and cKO neurons compared with that in WT neurons (Figure 4D). Similar impairments were also observed in cortical pyramidal neurons (Figure 4E–H). Together, these results show that *Brpf1* dosage is critical for dendrite arborization.

Neurological and neuropsychiatric disorders have been demonstrated to be highly associated with abnormalities in dendritic spines, which are small protrusions along dendrites and are typical postsynaptic structures in excitatory synapses (Harris, 1999; Penzes et al., 2011; Konopaske et al., 2014; Phillips and Pozzo-Miller, 2015). We explored whether there were any abnormalities in the dendritic spines. Examination of the number of spines per 10-μm length of the apical secondary dendritic branches in adult cortical pyramidal neurons demonstrated that *Brpf1* HTs exhibited a significant reduction in the number of spines compared to that in WT mice. *Brpf1* cKO mice showed a more severe decrease than that in *Brpf1* HT mice (Figure 4I–L). The regulation of spine morphology is a fundamental step in the establishment of functional neuronal networks (Penzes et al., 2011). Defects in spine morphology have been reported in various neurological and neuropsychiatric disorders (Nimchinsky et al., 2002; Lippman and Dunaevsky, 2005). We next assessed spine length after *Brpf1* disruption. As shown in Figure 4M, spine length was observed to be longer in *Brpf1* HTs than in WTs, and cKO spines were even longer than HT spines. Statistical analysis revealed a significant increase in spine length in *Brpf1* HT and cKO mice. Collectively, these data demonstrate that *Brpf1* dosage plays an important role in dendritic branching and spine morphology.

### *In vitro* Cell Culture Shows Impaired Both Dendritic and Axonal Development

**Figure 5:**
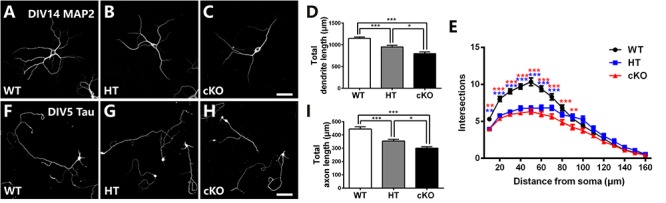
*Brpf1* haploinsufficiency suppresses dendritic and axonal outgrowth in *in vitro* cell culture. **(A–C)** Representative fluorescent images of cultured cells immunostained with anti-MAP2 antibodies from *Brpf1* WT **(A)** and HT **(B)**, and cKO **(C)** mice. Primary neurons were isolated from the E16.5 cerebral cortex and cultured for 14 days. Dendritic morphology was examined by immunostaining with a MAP2 antibody. **(D)** The quantification of total dendritic length reveals that *Brpf1* haploinsufficiency inhibits dendritic arborization *in vitro* (WT: *n* = 75; HT: *n* = 47; cKO: *n* = 49; one-way ANOVA (*F*_(2,168)_ = 27.09, *P* < 0.0001) with Bonferroni *post hoc* test: *P* = 0.0003 between WT and HT, *P* < 0.0001 between WT and cKO, *P* = 0.0134 between HT and cKO). **(E)** Sholl analysis shows reduced dendritic complexity of cultured neurons from *Brpf1* HT and cKO mice *in vitro* [WT: *n* = 52; HT: *n* = 47; cKO: *n* = 49; two-way ANOVA (effect of genotype: *P* < 0.0001) with Bonferroni *post hoc* test: for WT and HT, *P* = 0.0055 at 10 μm, *P* < 0.0001 at 20–70 μm from the cell body (blue asterisk), for WT and cKO, *P* = 0.0042 at 10 μm, *P* < 0.0001 at 20–80 μm, *P* = 0.0093 at 90 μm from the cell body (red asterisk)]. **(F–H)** Representative fluorescent images of 5 DIV cultured cells immunostained with anti-Tau antibodies from *Brpf1* WT **(F)** and HT **(G)**, and cKO **(H)** mice. **(I)** The quantification of axonal length demonstrates that decreased axonal length is observed in *Brpf1* HT and cKO mice *in vitro* (WT: *n* = 94; HT: *n* = 105; cKO: *n* = 63; one-way ANOVA (*F*_(2,259)_ = 22.00, *P* < 0.0001) with Bonferroni *post hoc* test: *P* < 0.0001 between WT and HT, WT and cKO, *P* = 0.048 between HT and cKO). Scale bar: **(A–C)**: 50 μm; **(F–H)**: 100 μm.

To further determine the defects in neurites, primary cell culture was carried out as previously reported (Yu et al., 2014). Cortical neurons were dissociated from E16.5 brains, cultured for 14 days and immunostained with anti-MAP2, a specific marker for dendrites. Neurons were then randomly picked up for analysis. As shown in Figure 5A–D, *Brpf1* WT neurons were well developed with numerous dendritic branches, whereas *Brpf1* HT neurons showed a 17% decrease in the total length of the dendritic branches compared with that of WT neurons. In particular, *Brpf1* cKO neurons displayed a significant decrease in the length of the dendritic branches compared with that of HT and WT neurons (Figure 5D). Sholl analysis exhibited a remarkable decrease in the number of intersections for *Brpf1* HT and cKO neurons (Figure 5E). Together, these data show that dendritic complexity is reduced in a *Brpf1* dosage-dependent manner *in vitro*, which is consistent with the alterations observed *in vivo*. These findings also demonstrate that the morphological changes observed *in vivo* are cell intrinsic rather than a consequence of altered behavior.

The axon length was then measured via immunostaining for Tau, an axonal marker, at 5 DIV. As shown in Figure 5F, *Brpf1* WT cells had considerably long axons, whereas axonal elongation in HT cells was impaired, with a 20.3% decrease in length (Figure 5G,I). In addition, *Brpf1* cKO cells exhibited noticeably underdeveloped axons with much shorter lengths compared to those of WT and HT cells (Figure 5I). In summary, we found that axonal growth was also obviously affected by *Brpf1* haploinsufficiency. Together, we concluded that *Brpf1* dosage is critical for proper dendritic and axonal outgrowth *in vitro*.

### Abnormal Formation of Synapses After *Brpf1* Deletion

**Figure 6:**
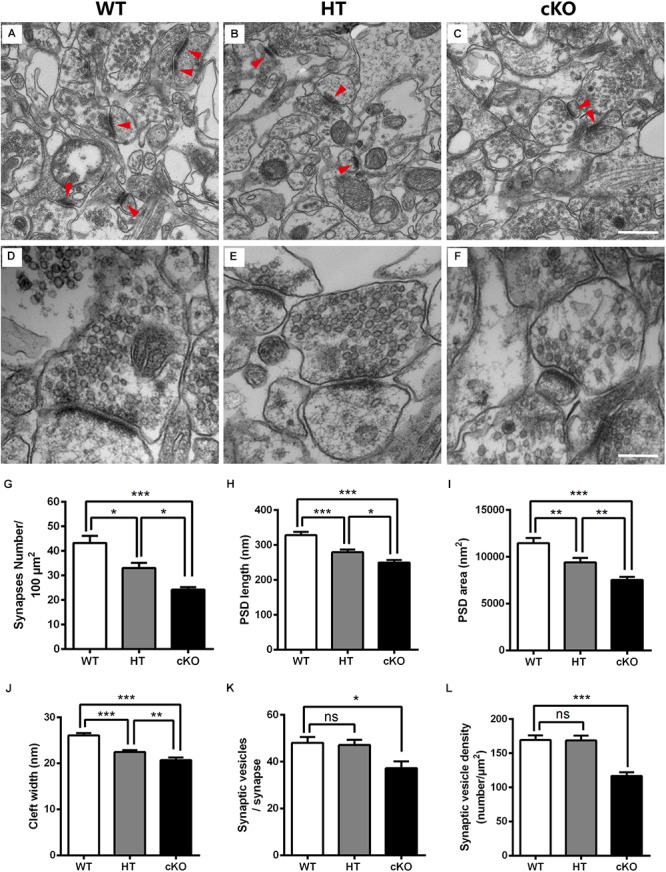
Electron microscopy shows the abnormal ultrastructure of synapses in *Brpf1* HT and cKO mice. **(A–C)** Representative low magnification (40,000x) images of hippocampal CA3 synapses are used to quantify synaptic density from *Brpf1* WT **(A)** and HT **(B)**, and cKO **(C)** mice. Excitatory glutamatergic synapses are asymmetric profiles with an apparent thick strongly labeled postsynaptic density (PSD) facing a number of synaptic vesicles (indicated with red arrowhead). **(D–F)** Representative high magnification (100,000x) electron micrographs were used to quantify PSD length and area and synaptic cleft width in *Brpf1* WT **(D)**, HT **(E)**, and cKO **(F)** mice. **(G)** Quantitative analysis of synapse density [WT: *n* = 4 brains; HT: *n* = 4 brains; cKO: *n* = 4 brains; one-way ANOVA (*F*_(2,9)_ = 20.14, *P* = 0.0005) with Bonferroni *post hoc* test: *P* = 0.0241 between WT and HT, *P* = 0.0004 between WT and cKO, *P* = 0.0484 between HT and cKO]. **(H)** Quantitative analysis of PSD length [WT: *n* = 72 synapses; HT: *n* = 77 synapses; cKO: *n* = 79 synapses; one-way ANOVA (*F*_(2,225)_ = 23.09, *P* < 0.0001) with Bonferroni *post hoc* test: *P* = 0.0001 between WT and HT, *P* < 0.0001 between WT and cKO, *P* = 0.0299 between HT and cKO]. PSDs are shorter in *Brpf1* HT and cKO synapses. **(I)** Quantitative analysis of PSD area [WT: *n* = 80 synapses; HT: *n* = 72 synapses; cKO: *n* = 72 synapses; one-way ANOVA (*F*_(2,221)_ = 18.89, *P* < 0.0001) with Bonferroni *post hoc* test: *P* = 0.0045 between WT and HT, *P* < 0.0001 between WT and cKO, *P* = 0.0095 between HT and cKO]. **(J)** Quantitative analysis of synaptic cleft width [WT: *n* = 67 synapses; HT: *n* = 74 synapses; cKO: *n* = 57 synapses; one-way ANOVA (*F*_(2,195)_ = 29.61, *P* < 0.0001) with Bonferroni *post hoc* test: *P* < 0.0001 between WT and HT, WT and cKO, *P* = 0.0087 between HT and cKO]. Synaptic clefts were thinner in *Brpf1* HT and cKO synapses. **(K)** The mean number of synaptic vesicles per synapse is not altered in *Brpf1* HTs but is decreased in *Brpf1* cKOs [WT: *n* = 72 synapses; HT: *n* = 69 synapses; cKO: *n* = 44 synapses; one-way ANOVA (*F*_(2,182)_ = 4.718, *P* = 0.0101) with Bonferroni *post hoc* test: *P* = 0.0133 between WT and cKO]. **(L)** The quantification of synaptic vesicle density (number/μm^2^) [WT: *n* = 72 synapses; HT: *n* = 69 synapses; cKO: *n* = 49 synapses; one-way ANOVA (*F*_(2,187)_ = 17.21, *P* < 0.0001) with Bonferroni *post hoc* test: *P* < 0.0001 between WT and cKO]. Scale bar: **(A–C)**: 500 nm; **(D–F)**: 200 nm.

Given the decreased number of dendritic spines and their abnormal morphology, we next conducted electron microscopy (EM) to detect the ultrastructure of excitatory synapses in the hippocampal CA3 region. The excitatory glutamatergic synapses exhibited asymmetric profiles with noticeably thick, strongly labeled postsynaptic density (PSD) facing a number of synaptic vesicles (Palay, 1956; Gray, 1959; Tao et al., 2012; Figure 6A–C). Under lower magnification, the number of synapses per 100-μm^2^ area was counted. The synaptic density was significantly reduced (by 23.5%) in *Brpf1* HTs compared to WTs, which was consistent with the reduced number of spines (Figure 4L, 6G). *Brpf1* cKO mice also exhibited a sharply decreased synaptic density (Figure 6G). Higher magnification views were used to quantify PSD area, and length and synaptic cleft width (Figure 6D–F). The postsynaptic terminal of excitatory synapses is characterized by PSD, which consists of a variety of scaffolding proteins, receptors and intracellular signaling molecules (Kaufmann and Moser, 2000; Newpher and Ehlers, 2009; Sala and Segal, 2014; Woolfrey and Srivastava, 2016). The PSD is thought to be associated with the formation of spines, which is altered in many neuropsychiatry diseases (Lamprecht et al., 2006). We found a 14.8% decrease in PSD length and a 17.9% decrease in PSD area in *Brpf1* HTs (Figure 6H,I). It has been reported that synaptic cleft structure is related to the stabilization of synapse formation and synaptic transmission (Rusakov and Kullmann, 1998; Nicholson, 2005; Dalva et al., 2007; Jung et al., 2017). Decreased synaptic cleft width was also observed (Figure 6J). The decrease observed in *Brpf1* cKOs was also dosage-dependent (Figure 6H–J).

Brain function depends critically on the ability of neurons to communicate with one another via neurotransmitters that are released from presynaptic terminals by Ca^2+^-triggered synaptic vesicle exocytosis (Sudhof, 2004; Sudhof and Rizo, 2011). Thus, we wondered whether any alterations in synaptic vesicle development contribute to the deficits in learning and memory and excitatory synaptic transmission detected after *Brpf1* disruption. No dramatic changes in the number of synaptic vesicles per single synapse or the density of synaptic vesicles were detected between *Brpf1* WTs and HTs. However, *Brpf1* cKO neurons exhibited a significant reduction in the number and density of synaptic vesicles (Figure 6K,L). Together, our data show that *Brpf1* haploinsufficiency results in the abnormal morphology of excitatory synapses, specifically reduced PSD length and cleft width, which may lead to reduced excitatory synaptic transmission.

## Discussion

In this study, we showed that *Brpf1* haploinsufficiency led to reduced dendritic complexity and the abnormal formation of synapses, which further resulted in decreased excitatory synaptic transmission. *Brpf1* HTs exhibited reduced anxiety and defective learning and memory. In addition, we demonstrate that neuronal alteration were similar between different brain regions and different types of neurons, including hippocampus granule cells and pyramidal neurons in the neocortex, which further confirm the important role of *Brpf1* dosage in neural circuit formation during cortical development. Our study provides new insights into the mechanisms underlying *Brpf1*-related disorders.

### *Brpf1* HTs Mimic ID to Some Extent

ID is a common neurodevelopmental disorder characterized by limitations in intellectual ability and impaired adaptive function. Recently, *Brpf1* has been identified as a candidate gene for ID (Mattioli et al., 2017; Yan et al., 2017). Patients with heterozygous *BRPF1* mutations suffer from infantile hypotonia, ID and language impairment. Here, we found that *Brpf1* HTs show impaired learning and memory and decreased levels of anxiety-related behavior. The behavioral phenotypes of *Brpf1* HTs recapitulate the deficits observed in individuals with *BRPF1* mutations and may act as a useful animal model for better elucidating the mechanism underlying *Brpf1*-haploinsufficiency-related neurodevelopmental disorders. Noteworthy since a portion of neurons in the amygdala are generated from Emx1-expressing progenitors in the dorsal telencephalon, these neurons may also contribute to the decreased levels of anxiety-related behavior.

### *Brpf1* Haploinsufficiency Affects Dendritic Arborization and Axonal Elongation

Deficiencies in the architecture of dendrites have been observed in a variety of neurodevelopmental and neuropsychiatric disorders, such as Rett syndrome, Down Syndrome and ID (Huttenlocher, 1974; Kulkarni and Firestein, 2012; Zoghbi and Bear, 2012). In this study, we showed that *Brpf1* haploinsufficiency in mice leads to reduced dendritic complexity in both cortical and hippocampal neurons. Consistent results were found in primary cultured neurons. These findings indicate that *Brpf1* dosage plays an important role in dendritic branching. Moreover, we found that axonal elongation is disrupted in a dosage-dependent manner. These findings suggest that *Brpf1* dosage contributes to neural circuit formation.

### *Brpf1* Regulates Synapse Morphogenesis

Previous studies have demonstrated that alterations in spine number and morphology are associated with changes in synaptic strength, neuronal activity and impaired learning and memory, which are observed in various neurodevelopmental disorders, such as Autism Spectrum Disorder (ASD) and ID (Hutsler and Zhang, 2010; Konopaske et al., 2014; Tang et al., 2014; Wang et al., 2017). In our study, we showed that *Brpf1* haploinsufficiency has a dramatic effect on spine morphology and synapse structure. Decreased spine density and increased spine length in *Brpf1* HTs likely lead to impairments in synaptic development and function. Moreover, *Brpf1* haploinsufficiency also causes ultrastructural alterations in synapses. Reductions in the depth of both the PSDs and the synaptic cleft were detected. These observations indicate the destabilization of these elements caused by *Brpf1* haploinsufficiency. Furthermore, we found that abnormalities in dendritic and spine development are functionally relevant, given that *Brpf1* haploinsufficiency results in reduced excitatory synaptic transmission. These observations indicate that *Brpf1* dosage is involved in spine formation and synapse morphogenesis.

Mammalian BRPF1 interacts with three HATs, MOZ, MORF, and HBO1, to form complexes that function as scaffolds to bridge subunit interactions, stimulate acetyltransferase activity and restrict substrate specificity (Doyon et al., 2006; Ullah et al., 2008; Lalonde et al., 2013). Previous studies have shown that *BRPF1* mutations in humans lead to decreased H3K23 acetylation (Mattioli et al., 2017; Yan et al., 2017). Similar defects were detected in the dorsal cortex of *Brpf1* knockout mice. It has been reported that the HATs CBP/p300 and P/CAF are required for the hyperacetylation-induced increase in neurite growth. Knockdown of CBP/p300 or P/CAF results in decreased neurite growth in cultured cerebellar granule neurons (Gaub et al., 2010). Future studies on the genome-wide changes in acetylation levels after *Brpf1* deletion and the interaction of the gene with other chromatin regulators will provide new insights into the pathology of *BRPF1*-related ID.

## Data Availability

The raw data supporting the conclusions of this manuscript will be made available by the authors, without undue reservation, to any qualified researcher.

## Ethics Statement

All animals were bred in the animal facility at Southeast University. All experiments were performed according to the approved guidelines of Southeast University.

## Author Contributions

CZ conceptualized the study. YS, JL, and CZ designed the experiments. YS performed the immunostaining, Golgi staining, and behavioral experiments. BY performed the electrophysiology experiments. BY and YS performed the electron microscope experiments. RB and YS performed the cell culture assay. CZ and YS analyzed the data and wrote the manuscript.

## Conflict of Interest Statement

The authors declare that the research was conducted in the absence of any commercial or financial relationships that could be construed as a potential conflict of interest.

## Supplementary Material

FIGURE S1 *Brpf1* HTs show a slightly thinner corpus callosum. **(A–B)** HE staining of serial brain sections. Three rostral to caudal coronal sections were prepared from *Brpf1* WT **(A–A′′)** and HT **(B–B′′)** brains and stained with HE to analyze the morphology of the corpus callosum at different planes. Scale bar: 0.5 mm.
