# Supplementary figures and images for: Brpf1 Haploinsufficiency Impairs Dendritic Arborization and Spine Formation, Leading to Cognitive Deficits
Source: Front Cell Neurosci. 2019 Jun 4;13:249. doi: 10.3389/fncel.2019.00249 (PMC6558182; doi:10.3389/fncel.2019.00249)

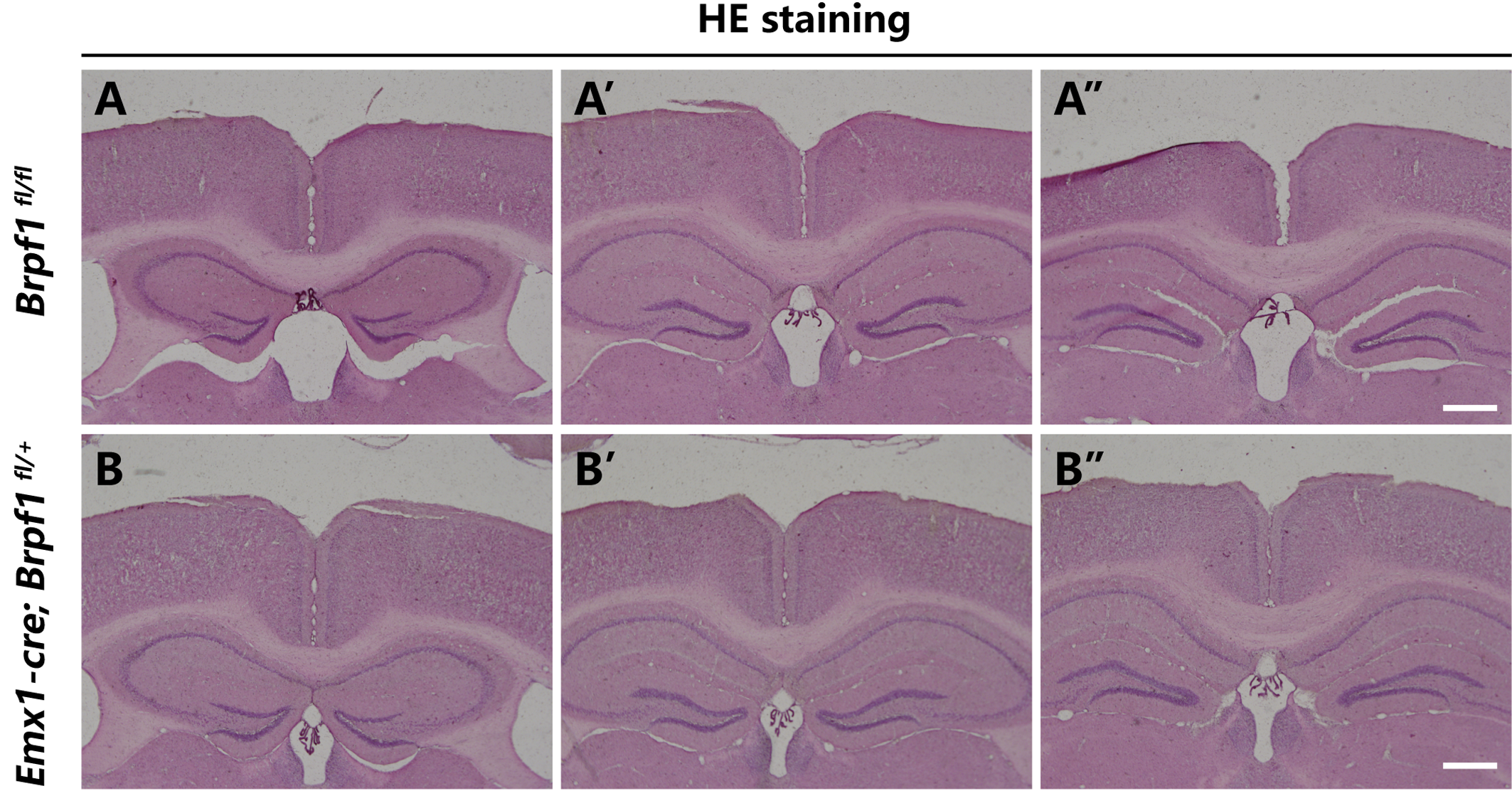

Supplement: FIGURE S1 — Brpf1 HTs show a slightly thinner corpus callosum. (A–B) HE staining of serial brain sections. Three rostral to caudal coronal sections were prepared from Brpf1 WT (A–A′′) and HT (B–B′′) brains and stained with HE to analyze the morphology of the corpus callosum at different planes. Scale bar: 0.5 mm. [file Image_1.TIF]
